# Effect of a 12-week submaximal swimming training in rats exposed to tobacco- derived nitrosamine ketone

**DOI:** 10.22088/cjim.9.2.158

**Published:** 2018

**Authors:** Ali Barzegari, Shadmehr Mirdar

**Affiliations:** 1 Department of Physical Education and Sports, Payam Noor University, Tehran, Iran.; 2 Department of Exercise Physiology, Faculty of Sport Sciences, University of Mazandaran, Mazandaran, Iran.

**Keywords:** Interleukin 10, Tobacco-derived nitrosamine ketone, Submaximal aerobic activity, Inflammation.

## Abstract

**Background:**

Tobacco contains carcinogens such as NNK (nicotine-derived nitrosamine ketone) that makes induction of lung cancer by changing the stimulation of IL-10 expression. The aim of this study was to investigate changes in resting levels of IL-10 in lung tissues of rats exposed to NNK after a 12-week aerobic submaximal swimming training.

**Methods:**

For this purpose, 46 Wistar rats were randomly divided into five groups consisting training, training + NNK, NNK, saline and control. NNK-induced groups received NNK subcutaneously one day per week at a rate of 12/5 mg per kg body weight and the training groups performed submaximal swimming training for 12 weeks. The levels of IL-10 in homogenized lung tissue were measured by ELISA.

**Results:**

Findings indicated that a period of swimming training increased the IL-10 levels significantly in lung tissue of training group when compared to control (P=0.00) and NNK groups (P=0.00). Also, a significant increase of IL-10 level was observed in exercise + NNK group when compared to NNK group (p≤0/02). Furthermore, it was observed that IL-10 levels of NNK group had a significant decrease when compared to training group (P=0/00), training + NNK group (p≤0/02), but had insignificant increase when compared to saline group (p≤0/85).

**Conclusions:**

Generally, it could be confirmed that regular submaximal aerobic training plays an important role in the inhibition of the effects of lung inflammation induced by NNK via increasing IL-10 activity.

Lung cancer is the third most common cancer after prostate gland and breast cancer (1) is the leading cause of death worldwide, accounting for 1.59 million deaths in 2012 (2). Thankappan and Thresia reported that there had been 5 million deaths per year in the world; including 2.41 million deaths in the developing countries and 2.43 million in the developed countries, only because of tobacco consumption. Tobacco smoking is a well-established risk factor for human lung cancer (3). More than 85% of all lung cancers are linked to tobacco smoke (4). Tobacco smoke contains at least 55 compounds known-inducing tumors in laboratory animals. One of these carcinogens, 4-(methylnitrosamino)-1-(3-pyridyl)-1-butanone Nicotine-derived nitrosamine ketone (NNK), being potent and abundant, is remarkable in the lungs of rodents (3). Excessive inhaling of nicotine results in the alteration of signalling pathways responsible for proliferation, apoptosis and metastasis. Lung cancer culminates the series of changes in the signalling pathways. One of the changes includes desensitization of its cognate receptor, nicotinic acetylcholine receptor (nAChRs) which is a heterogeneous ligandgated ion channel receptor expressed in numerous tissues including lung tissue (5). 

On the other hand, alveolar macrophage can produce anti-inflammatory cytokines such as IL-10 which inhibit or control inflammation. The suppression of TNF-𝛼 function favors tumor proliferation and differentiation through the activation of IL-10 (6). Several reports have indicated that IL-10 promoter polymorphisms are related with various cancer risks including lung cancer (7). Further studies showed that IL-10 promoter polymorphisms were correlated with tumor progression and clinical outcomes in a number of cancers, such as breast cancer, squamous cell carcinoma (SCC) after renal transplantation, and non-small cell lung cancer (NSCLC) (8). SCC is predominantly associated with cigarette smoking, and induction of IL-10 has been reported in response to cigarette smoke extracts and nicotine-derived nitrosamine ketone (NNK), a major carcinogenic compound of cigarette smoke extract(9).

Furthermore, NNK inhibits the production of IL-12, TNF and NO, but stimulates IL-10 and PGE2 release (10). Lipopolysaccharide-elicited chronic lung inflammation significantly increases the risk of NNK-mediated lung tumorigenesis, and chronic extrinsic lung inflammation induced by NNK enhancing lung tumorigenesis in mice (11). Chronic inflammation may promote neoplasia by inducing preneoplastic mutation, cell proliferation, and resistance to apoptosis, invasiveness, angiogenesis, and secretion of immune suppressive factors (12).

As lung cancer is one of the most common causes of death, it can be treatable if diagnosed soon (13). In NSCLC, IL-10 overproduction at the tumor site has been implicated in tumor-mediated immunosuppression, enhanced angiogenesis and serum IL-10 appears to be an indicator of poor prognosis (14). In this regard, modified factors such as physical activity can help us to prevent and treat lung cancer by modulating the inflammatory process. Aerobic exercise is a powerful tool to combat oxidative stress activation; it also provides a protective mechanism that helps to re-establish cellular homeostasis, decreases the release of pro-inflammatory cytokines, and improves immune responses (15). Demarzo et al. in mentioned the anti-inflammatory and anti-cell proliferation effect of prolonged swimming exercise with moderate intensity in colon cancer (16). Additionally, evidence indicates that physical activity may reduce the risk of lung cancer with several possible mechanisms (17). In preclinical studies, an 8-week moderate swimming intervention in mice injected with DMBA decreased quantities of lymphocytes (and macrophages) expressing IL-10 compared with sedentary mice (18). As Leonardo et al. (2015) reported, swimming sessions resulted in an increase in the IL-10 levels (15). So, given the importance of prevention of lung cancer, and also the lack of necessary and adequate information regarding the impact of exercise on IL-10 levels in lung tissue exposed to NNK in tobacco, the aim of this study was to examine the effects of submaximal swimming on IL-10 Wistar rats exposed to NNK.

## Methods

**Ethical approval: **All procedures of this study were in the accordance of the Guide for the Care and Use of Laboratory Animals published by the US National Institutes of Health (NIH Publication, 8th Edition, 2011). This study was approved by HRI Ethics Committee of Babol University of Medical Sciences (Code No: MUBABOL.HRI.REC.1395.109).

**Experimental design: **The experimental design was performed in Mazandaran University. 46 Wistar rats with an average weight of 105.84±27/93 grams were purchased from Pasteur Institute of Iran. After two weeks of becoming familiar with the environment and research protocol, the animals were divided randomly into 5 groups consisting of training group (n=10), NNK (n=10), training + NNK (n=10), saline (n=10) and control (n=6). Animals in an environment with an average temperature of 22 ± 1/4 ° C, humidity 55% and the light dark cycle of 12:12 h were kept in cages made of polycarbonate. Keeping animals was carried out in accordance with the international health institute instructions, as well as the protocol of this study was performed in accordance with the Declaration of Helsinki as a statement of ethical principles for medical research. Animals consumed pellets and water freely. NNK is injected subcutaneously once a week at a rate of 12/5 (mg/kg/bw) for 12 weeks and also the solvent group received distilled water (16). 

Rats in training groups were familiarized with water and swimming in the pool with dimensions of 50 × 50 × 100 cm by temperature of 30-32° C for one week (5 days) and 20 minutes each time. The power of water during swimming was 4 to 10 liter/min for 25 to 60 min, 5 times a week; (table 1). To eliminate the effect of acute exercise, sampling of the animals was performed 48 hours after the last session of swimming. Then the animals were anesthetized using intraperitoneal injection of ketamine (30-50 mg/kg) and xylazine (3-5 mg/kg). After thoracic surgery, lung tissue was isolated and placed in the microtubes then frizzed in liquid nitrogen at -70°C. To prepare the laboratory analysis, 100 mg of lung tissue with 1 ml of PBS buffer 100 mM was homogenized and centrifuged for 15 minutes at 6000 rpm. Then the obtained supernatant was transported to the laboratory for measuring IL-10. IL-10 was measured and quantified using ELISA based on the kit’s instructions. BE53101 ELISA kit manufactured by German Company IBL International GmbH was used to measure IL-10.

**Statistical analysis: **To determine the normal distribution of data and homogeneity of variance, we used Shapiro-Wilk test and Leven's test, respectively. The mean and standard deviation were used to report the values of measured variable. All statistical analyses were performed using the SPSS statistical software program (Version 24.0).

## Results

Table 2 shows the mean and standard deviation of rats’ weights in several research groups. Results of ANOVA test did not show significant changes in body weight of different research groups.

**Table 1:** Mean and standard deviation of weight average in different research groups

**Weight (mean±SD)**	
**Group**	
Control	11.17±5.210
Saline	23.24±8.208
Training+NNK	88.61±3.274
NNK	48.70±6.285
Training	38.54±8.262

The results of ANOVA test showed that the levels of IL-10 in lung tissue of research groups had a significant difference (P= 0.00). Furthermore, the result of post hoc test showed that levels of IL-10 were higher in training group comparing with the control (175.45%), NNK (162.79%), saline (240.53%) and training + NNK groups (53.93%) (P=0.00).

**Figure 1:**
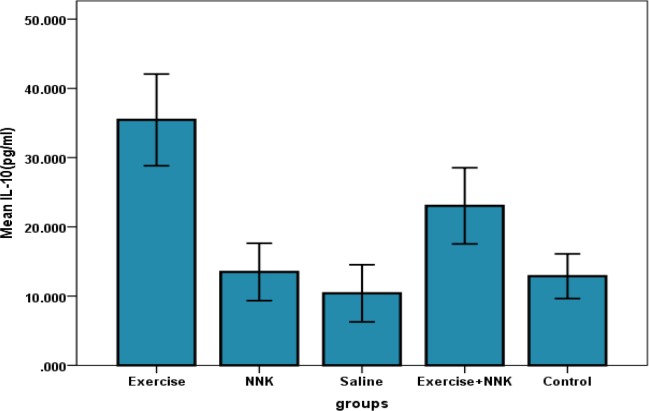
Changes in IL-10 values (pg/ml) in lung tissue of research groups

In addition, findings noted that IL-10 levels in lung tissue of NNK group decreased significantly when compared to the training (61/95%) and, training + NNK groups (41/42%) (P=0.00, P=0.02 respectively), while these differences were not significant when compared to the saline and control groups (respectively: P=0.85, P=1.00). Moreover, it showed that intervention training with induction of NNK resulted in significant decrease in IL-10 levels of lung tissue (35.03%) when compared to training group (P= 0.00), but increased significantly when compared to NNK (70.72%), control (78.94%) and saline groups (121.23%) (respectively P=0.02, P=0.05 and P=0.00) (figure 1).

* Signs of significant changes compared to control, # signs of significant changes compared to saline, ¥ signs of significant changes compared to training, £ signs of significant changes compared to NNK, € signs of significant changes compared to NNK + training

## Discussion

In the present study, the effect of submaximal swimming training on resting levels of IL-10 in lung tissue of rats exposed to tobacco-derived nitrosamine ketone was examined. The results showed that 12 weeks swimming training significantly decreased the mean levels of IL-10 in lung tissue of rats in swimming training group compared to the controls. Consistent with our findings, Kesherwani et al. reported increased levels of IL-10 after a long period of swimming exercise training (21). The release of IL-10 and its circulation after exercise also contributes to mediation of the anti-inflammatory effects of exercise. IL-10 acts as an anti-inflammatory molecule that has been primarily suggested by studies showing inhibition of the synthesis of a large spectrum of proinflammatory cytokines with different cells. Thus IL-10 inhibits the production of IL-1α, IL-1β, and TNF-α as well as the production of chemokines, including IL-8. These cytokines and chemokines play a critical role in the activation of granulocytes, monocytes/macrophages, natural killer cells, T and B cells and in their recruitment to the sites of inflammation (22). Wang and et al. suggested that IL-10 plays an important role in orchestrating the inflammatory reaction involving macrophage/monocyte activation in particular. Addition of IL-10 to lipopolysaccharide stimulated human mononuclear cells and neutrophils suppressed cytokine synthesis, mainly via the inhibition of the transcription of their corresponding genes (22). IL-10 also prevents cytokine synthesis by post transcriptional mechanisms, as shown in human macrophages where the inhibition of proinflammatory cytokines is a direct consequence of mRNA degradation of their corresponding genes (22). 

Moreover Moore et al. stated that IL-10 modulates the expression of some cytokines, soluble mediators and cell surface molecules by cells of myeloid origin, influencing the ability to activate and sustain immune and inflammatory responses (22). Furthermore, Vieira et al. demonstrated that in sensitized animals, aerobic exercise training increased the expression of IL-10 by inflammatory cells, representing a possible mechanism of exercise-induced decrease in allergic inflammation (23). In several studies, the results were inconsistent or conflicting. In this regard, Bizheh et al. (2014) demonstrated that 8 weeks of aerobic training in water reduces the IL-10 levels significantly. Actually, it can be concluded that a reduction in the levels of IL-10 that occurs with exercise is different from the decline that takes place as a result of disease recurrence (24). The possible reasons for inconsistent findings of this study with other researchers' findings can be noted on the nature and duration of the exercise. Furthermore, another reason may be related to the characteristics of subjects, as Bizheh et al. investigated the MS patients (24).

IL-10 levels decreased significantly in lung tissue of NNK group when compared to the training groups, while they increased insignificantly as compared to saline and control groups. The results of the present study are consistent with other researchers reporting that exposure to NNK contributes to the stimulation of IL-10 levels (4, 25). Proulx et al. reported that NNK stimulated the alveolar macrophage PGE2 production and exogenous PGE2 stimulated IL-10 production. Therefore, the increasing IL-10 release might be mediated with the stimulation of PGE2 production by NNK. They also demonstrated that the stimulation of alveolar macrophages PGE2 production by NNK was mediated by COX-2. NNK stimulated COX-2 expression leading to an upregulation of PGE2 production, which in turn may upregulate IL-10 release via alveolar macrophages. In the presence of PGE2, which stimulates IL-10 production, we can suggest that there is a different pathway implicated in the inhibitory effect of NNK on alveolar macrophages IL-10 production (26). Therriault et al. also noted that IL-10 is an anti-inflammatory cytokine that inhibits various macrophage functions and production of proinflammatory cytokines and chemokines. IL-10 is a potent inhibitor of both the alveolar macrophages and NK-cell anti-tumor activities. 

In addition to being produced by tumor cells, the increased alveolar macrophages IL-10 production induced by NNK may favor the development of lung cancer. However, NNK did not modulate the production of TGF-β, which was involved in tumor growth (4). Probably, the cause of insignificant changes of IL-10 in our study may be related to duration and doses of NNK injection. As, Therriault et al. reported, the low dose of NNK (1.5 mg/ml) given once does not significantly increase alveolar macrophages IL-10 production, but the same concentration given every day for 5 days (a total of 7.5 mg/ml) causes a 2.8-fold increase of IL-10 release, which is higher than the 1.6- fold increase observed at 207 mg/ml after 20 h of treatment (4). 

In the present study, we also demonstrated that 12 weeks swimming exercise inhibits lung inflammation and significantly enhanced IL-10 release in lung tissue in an experimental model of NNK-induced lung inflammation. It is well-established that several cytokines have different roles in cancer and during prolonged-exercise. IL-10 was expected to increase in both with or without chemotherapy. However, Pedrinolla et al. found an increase in IL-10 only in chemo subjects. This might be due to the different roles of this cytokine in different situations. In fact in a cancer-environment, IL-10 would act like anti-apoptotic and tumor-growth factor, but during exercise it would act like anti-inflammatory cytokine, increasing the cortisol-level and inhibiting TNF-α (26). 

Consistently, Gonçalves et al.observed an effect of both lipopolysaccharide (LPS)-induced acute lung injury and exercise on the release of IL-6 which is one of the first acute phase cytokines released in sepsis/endotoxemia and is followed by the increases in the levels of IL-10. IL-6 has been shown to stimulate the production of IL-10, a cytokine with well-known anti-inflammatory effects. They further observed a negative association between these two anti-inflammatory cytokines and the density of neutrophils in lung tissue (27). In Toledo et al.’s study, the smoke-induced reduction in IL-10 was inhibited by exercise training. IL-10 modulates the expression of some cytokines, soluble mediators and cell surface molecules by cells in myeloid origin, influencing the ability to activate and sustain immune and inflammatory responses (28). Although, in this study many variables such as species, sex, weight, environmental factors (noise, light, humidity, and temperature), training factors (type, duration and intensity of exercise) and diet were under control, one of the limitations of this study, was that the overnight physical activity of the subjects was not under control and the intake of NNK in subjects was not measured.

In conclusion, we have shown that submaximal swimming training reduces oxidative stress and protects against the development of emphysema in rats. Exercise training inhibited the inflammatory development by a significant decrease in oxidative stress, reduction in ROS production in lung parenchyma, and an increase in IL-10 of the inflammatory cells in the alveolar wall. These results support the hypothesis that the antioxidant effect of exercise combats cellular oxidative stress, which is induced by NNK and has an important role in cancer development.
